# Inferring reward prediction errors in patients with schizophrenia: a dynamic reward task for reinforcement learning

**DOI:** 10.3389/fpsyg.2014.01282

**Published:** 2014-11-11

**Authors:** Chia-Tzu Li, Wen-Sung Lai, Chih-Min Liu, Yung-Fong Hsu

**Affiliations:** ^1^Department of Psychology, National Taiwan UniversityTaipei, Taiwan; ^2^Graduate Institute of Brain and Mind Sciences, National Taiwan UniversityTaipei, Taiwan; ^3^Neurobiology and Cognitive Science Center, National Taiwan UniversityTaipei, Taiwan; ^4^Department of Psychiatry, National Taiwan University HospitalTaipei, Taiwan

**Keywords:** Bayesian estimation method, dynamic reward task, matching law, psychosis, reinforcement learning model, reward prediction error, schizophrenia

## Abstract

Abnormalities in the dopamine system have long been implicated in explanations of reinforcement learning and psychosis. The updated reward prediction error (RPE)—a discrepancy between the predicted and actual rewards—is thought to be encoded by dopaminergic neurons. Dysregulation of dopamine systems could alter the appraisal of stimuli and eventually lead to schizophrenia. Accordingly, the measurement of RPE provides a potential behavioral index for the evaluation of brain dopamine activity and psychotic symptoms. Here, we assess two features potentially crucial to the RPE process, namely belief formation and belief perseveration, via a probability learning task and reinforcement-learning modeling. Forty-five patients with schizophrenia [26 high-psychosis and 19 low-psychosis, based on their p1 and p3 scores in the positive-symptom subscales of the Positive and Negative Syndrome Scale (PANSS)] and 24 controls were tested in a feedback-based dynamic reward task for their RPE-related decision making. While task scores across the three groups were similar, matching law analysis revealed that the reward sensitivities of both psychosis groups were lower than that of controls. Trial-by-trial data were further fit with a reinforcement learning model using the Bayesian estimation approach. Model fitting results indicated that both psychosis groups tend to update their reward values more rapidly than controls. Moreover, among the three groups, high-psychosis patients had the lowest degree of choice perseveration. Lumping patients' data together, we also found that patients' perseveration appears to be negatively correlated (*p* = 0.09, trending toward significance) with their PANSS p1 + p3 scores. Our method provides an alternative for investigating reward-related learning and decision making in basic and clinical settings.

## Introduction

Many everyday decisions are made on the basis of experience but with incomplete knowledge or insufficient feedback. As such, making appropriate decisions requires the ability to update information about alternatives based on previous experiences. In the past decades, the study of reward-based decision making and action has attracted much attention. However, it remains unclear how decisions are made in patients with mental disorders. Interestingly, patients with schizophrenia (abbreviated “SZ patients” hereafter) have been found to display abnormalities in reward processing and deficits in reinforcement learning (Waltz et al., 2007; Gold et al., 2008; Murray et al., 2008). The Cognitive Neuroscience Treatment Research to Improve Cognition in Schizophrenia (CNTRICS) initiative, funded by the National Institute of Mental Health, U.S.A., selected “reinforcement learning” as one of the most promising functional imaging biomarkers for immediate translational development for use in research on long-term memory in SZ patients (Ragland et al., 2012). In the reinforcement learning literature, the reward prediction error (RPE)—a discrepancy between predicted and actual reward—is thought to play an important role in the value-updating process (Glimcher, 2011). Past studies have shown that midbrain dopamine neurons encode RPE during reinforcement learning (Schultz et al., 1997; Tobler et al., 2003; Bayer and Glimcher, 2005; Niv, 2009). Reinforcement learning behavior is also altered after the administration of dopaminergic drugs (Pessiglione et al., 2006; Rutledge et al., 2009).

According to the dopamine hypothesis of schizophrenia, psychotic symptoms, including hallucination and delusion, are caused by hyperactivity of the dopaminergic system in the midbrain (Carlsson and Carlsson, 1990; Seeman et al., 2005). Emerging evidence indicates that the firing of midbrain dopamine neurons appears to correlate with the history of reward delivery and RPE signals (Hollerman and Schultz, 1998; Bayer and Glimcher, 2005). Neuroimaging studies have further suggested that this RPE system might be disrupted in SZ patients or psychosis (Juckel et al., 2006; Corlett et al., 2007; Frank, 2008; Gold et al., 2008; Murray et al., 2008). These studies indicate that aberrant RPE processes encoded by dopamine neurons might link the abnormal physiological activities and subjective psychotic experiences reported by SZ patients (Fletcher and Frith, 2009; Corlett et al., 2010). In another line of research, Kapur et al. proposed that abnormalities in the dopamine system might alter the appraisal of stimuli and lead eventually to psychotic symptoms (Kapur, 2003; Kapur et al., 2005; Howes and Kapur, 2009; see also Miller, 1976). Thus, considering dopamine's role in RPE and in motivational salience^1^, psychosis might result from the disturbances in RPE signaling that are generated by the dopamine system, in which inferences and beliefs about the real world cannot be properly updated or corrected.

Further evidence correlating RPE with dopamine activity is provided by two recent studies using decision-making tasks and reinforcement-learning modeling in humans and in mice. Motivated by an earlier work by Frank et al. (2004) of the role of dopamine on RPE in Parkinson's patients, Rutledge et al. (2009) found that patients with Parkinson's disease, which is characterized by a deficit in dopamine neurons in the midbrain, increased their value-updating speed in a “dynamic foraging task” after L-DOPA (which is a direct precursor to dopamine) manipulation. Chen et al. (2012) reported that *Akt1* (which is one of the schizophrenia candidate genes and a downstream kinase for dopamine D2 receptors) mutant mice exhibit, on average, higher learning rates and lower degrees of exploitation than wild-type control mice in a “dynamic foraging T-maze.” Both studies indicate that the subjects give RPE signals greater weight and change their beliefs more frequently when their dopamine activity is increased.

The above studies suggest that through dopamine activity on RPE signaling, reinforcement learning involves a balance between updating (for belief formation) and exploitation (for belief perseveration). To examine this topic more closely, in the present study we recruited chronic SZ patients (and healthy controls) and adopted a feedback-based, computerized version of the *dynamic reward task* (DRT), modified from the “dynamic foraging task” of Rutledge et al. (2009) and the “dynamic foraging T-maze” of Chen et al. (2012). In this new version, subjects were instructed to choose between two decks of cards on the computer screen; each deck was assigned a different probability of reward. Importantly, the ratio of reward probabilities associated with each of the decks changed block by block without informing the subjects.

There are two advantages for using the DRT in this research. First, in the DRT, as the higher reward probability deck is alternated across blocks, it is necessary for subjects to have well-functioning RPEs to perform well in the task. In other words, the DRT is more sensitive for detecting abnormalities in RPEs than traditional “static” tasks that do not have the feature of changing reward probabilities. Second, probability learning also involves the process of belief formation, which is the subjective probability of specific events occurring. While both belief perseveration and belief formation are not RPE *per se*, in the DRT both processes can be inferred through RPE modeling. In particular, each individual data can be fit by a standard reinforcement learning model (Sutton and Barto, 1998) to characterize the reward learning process. Such a model allows one to (i) assess the speed of the value-updating process on a trial-by-trial basis, and (ii) evaluate each subject's overall degree of exploitation. A hierarchical Bayesian method that takes into account individual differences both between and within groups was used to estimate the parameters in the model.

We hypothesize that SZ patients exhibit higher learning rates and reduced exploitation compared with healthy controls and that these patterns are associated with the severity of the positive psychotic symptoms of the SZ patients. Since RPE signaling via dopamine plays a crucial role in reinforcement learning, we also hypothesize that in this task SZ patients are less adept at allocating their choice behavior in accord with the reward frequencies that they have experienced.

## Materials and methods

### Participants

Forty-five DSM-IV diagnosed SZ patients and 24 healthy controls aged between 18 and 65 years were recruited from the National Taiwan University Hospital, Taipei, Taiwan. The recruitment and experimental procedures followed ethical guidelines and were approved by the Review Board of the institution. Written informed consent was obtained from each participant before the experiment. All patients were chronic SZ patients; they were clinically stable, as determined by their psychiatrists, and were being treated with antipsychotic drugs. Furthermore, these SZ patients were free of mental retardation, epilepsy or other brain damage, mood disorders, schizoaffective disorder, and alcohol and drug abuse. The psychopathological symptoms of the patients were assessed by two well-trained psychiatrists using the Positive and Negative Syndrome Scale (PANSS) for schizophrenia (Kay et al., 1987).

Some evidence has shown that dysregulation of dopamine is linked to the positive symptoms of schizophrenia (Gradin et al., 2011; see also Corlett et al., 2007; Murray et al., 2008).^2^ Following Fletcher and Frith (2009) that RPE signaling in SZ patients is associated with abnormal perceptions (i.e., hallucinations) and abnormal beliefs (i.e., delusions), in this study we opted for SZ patients' score of the two positive-symptom subscales p1 “delusion” and p3 “hallucinatory behavior” in the PANSS as an index for the severity of their psychotic symptoms.^3^ A similar use of these two subscales for indexing the psychiatric symptoms also can be found in Gradin et al. (2011). Each item in the PANSS is based on a 7-point scale. A rating of 2 means the symptom is “minimal” and a patient's behavior may be at the upper extreme of being normal. A rating of 3 means “mild” that is indicative of a symptom whose presence is clearly established but not pronounced enough to interfere with day-to-day functioning (Kay et al., 1987). It is thus reasonable to use a cut-off of 3 for the splitting of the patient group. Specifically, for each SZ patient, if either the p1 or p3 score was equal to or greater than 3, then s/he was categorized into the high-psychosis group; otherwise, s/he was categorized into the low-psychosis group. Among the 45 SZ patients that we recruited in this study, 26 were categorized as high-psychosis patients, and the other 19 patients were in the low-psychosis group. The control group included 24 healthy subjects without any psychiatric DSM-IV axis-I or II disorders.

Demographic information for all participants is displayed in Table 1, from which one sees that age and gender, but not education level, were roughly matched across the three groups. Moreover, all PANSS subscores for the high-psychosis group were significantly higher than those for the low-psychosis group (all *p*s < 0.05). To evaluate the impact of drug dosage on SZ patients' performance, we also computed the averaged daily chlorpromazine-equivalent antipsychotic doses of the two psychosis groups as described previously (Woods, 2003). We found no significant difference [*t*_(43)_ = 0.94, *p* = 0.35] between the adjusted doses of the high-psychosis and low-psychosis groups (*M* ± *SD*: 325.38 ± 243.61 vs. 267.11 ± 134.96 mg).

**Table 1:** **Demographic information of the high-psychosis, low-psychosis, and control groups**.

	**Patients**				**Controls**			
	**High psychosis *N* = 26**		**Low psychosis *N* = 19**					
					***N* = 24**			
	**Mean**	**(*SD*)**	**Mean**	**(*SD*)**	**Mean**	**(*SD*)**		
Age	39.50	(11.70)	38.32	(12.87)	36.54	(10.10)	*F*_(2, 66)_ = 0.36	*p* = 0.70
Gender (M:F)	12:14		10:9		12:12			
Education (year)	13.65	(1.90)	13.95	(2.17)	15.08	(2.84)	*F*_(2, 66)_ = 3.39	*p* = 0.04
Age of onset	24.73	(8.03)	24.26	(9.95)			*t*_(43)_ = 0.45	*p* = 0.66
**MEDICATION (*N*)**								
Typical	4		1					
Atypical	18		17					
Combination	4		1					
**PANSS SCORE**								
Positive	13.69	(3.51)	8.63	(2.09)			*t*_(43)_ = 8.11	*p* < 0.01
Negative	16.12	(5.49)	12.84	(3.96)			*t*_(43)_ = 2.63	*p* = 0.01
General	29.46	(8.19)	22.37	(4.70)			*t*_(43)_ = 3.44	*p* < 0.01
p1 + p3	5.92	(1.70)	2.42	(0.69)			*t*_(43)_ = 8.48	*p* < 0.01
Total	63	(13.90)	46.95	(9.06)			*t*_(43)_ = 5.05	*p* < 0.01

### The dynamic reward task (DRT)

**Figure 1:**
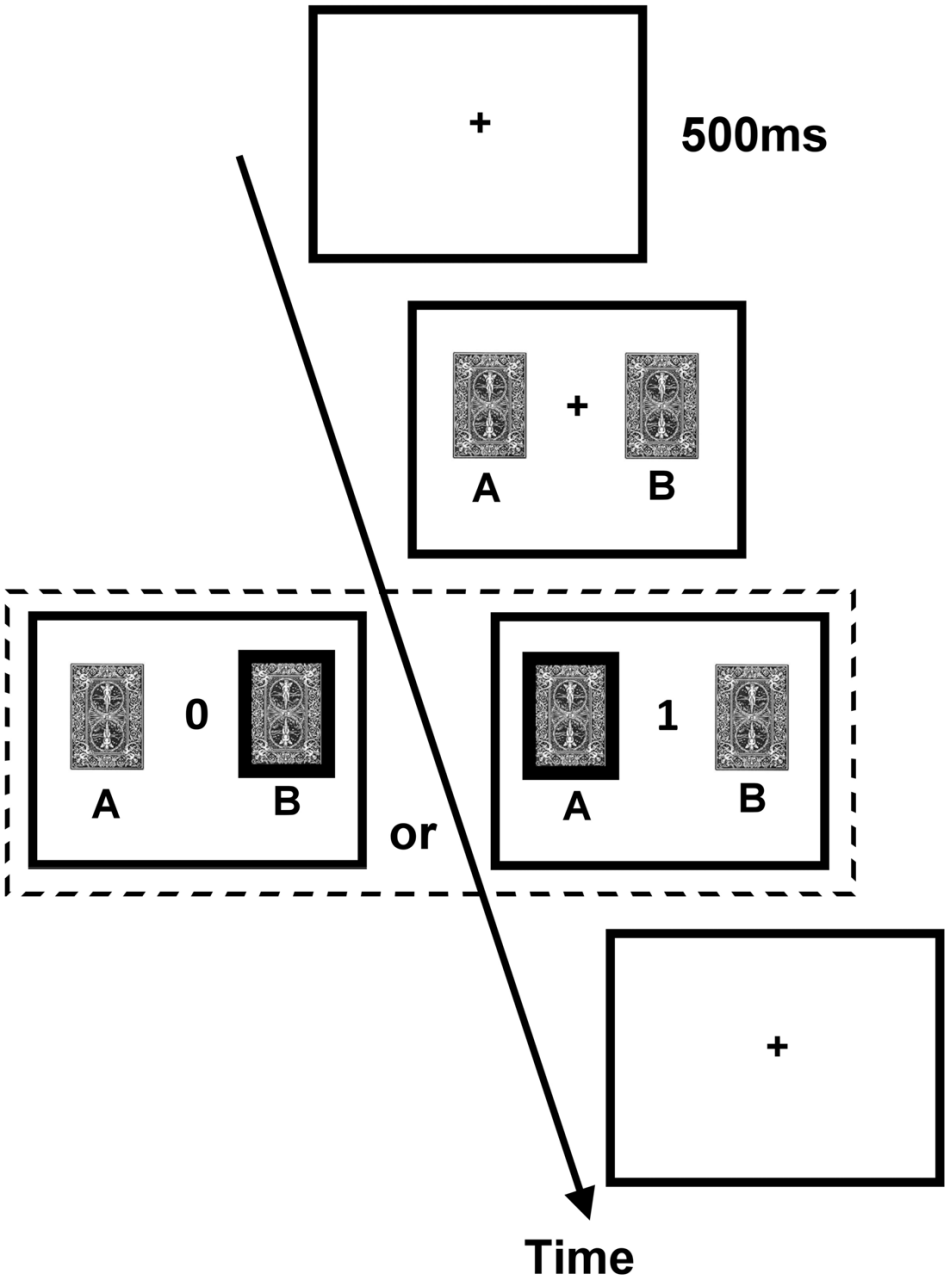
**Procedure of the dynamic reward task**. Each trial began with the presentation of the fixation cross (size 0.6° × 0.6° visual angle) for 500 ms. Subsequently, two identical cards (size 3.81° × 5.08° visual angle) appeared on the two sides of the fixation cross until a participant chose one card. After a card was chosen, the score of the chosen card (either 0 or 1; size 1.27° × 1.53° visual angle) was presented in the center of the screen until a participant pressed SPACE on the keyboard to end the trial and to initiate the next trial.

The DRT employed a trial-by-trial two-card scenario. The procedure of an exemplary trial is illustrated in Figure 1. On each trial, two cards, one drawn from deck A and the other from deck B, were presented side by side on the computer screen without showing the reward values until the subject made a choice between them. Next, feedback of either 0 (no reward) or 1 (reward) point was revealed to the subject in the center of the screen. The subject was instructed to maximize the total point, and monetary reward was given to him/her at the end of the experiment (one point = one New Taiwan dollar, which is about 0.033 US dollars). The ratio of reward probabilities of the two decks varied in a block design, and changes in blocks were not signaled to the subjects. Because the overall probabilities of reward assigned to the two decks were set higher than other similar (animal) studies of matching behavior and reinforcement learning (e.g., Corrado et al., 2005; Lau and Glimcher, 2005), we did not “bait” a card until the next time the subject chose it (i.e., the reward status of the non-chosen card was redefined on each trial). Furthermore, the DRT consisted of one training session and one testing session.

#### The training session

There were 40 trials in the training session. These trials were used to allow subjects to familiarize themselves with the experimental procedure and to learn that one of the two decks had a higher reward probability. The reward probability ratio of the two decks was 1:6, and the sum probability of gain across both cards was 0.6 (i.e., the two decks' probabilities of obtaining 1 point were 0.0857 and 0.5143, respectively).

#### The testing session

The same procedure was used in the testing session; each subject had to complete 480 trials, and was instructed to maximize the final score. There were 6 test blocks that contained 70–90 trials each, and the probabilistic structure was similar to that used in Rutledge et al. (2009). The two decks' gain ratios were 1:6, 6:1, 3:1, or 1:3; these ratios were constant within each block, and the overall probability of gain was fixed at 0.6. As shown in Table 2, two pseudorandom sequences^4^ of blocks were used in the testing session, and each subject was randomly assigned to one of the sequences. After the completion of one block, the deck with the higher reward probability became the deck with the lower reward probability, and another gain ratio was instated. Subjects were told that the advantageous deck might not always be the same deck and that they would receive monetary payment based on their total points.

**Table 2:** **Two sequences of probability assignment used in the testing session**.

**Block**	**1**	**2**	**3**	**4**	**5**	**6**
**SEQUENCE #1**						
Deck A	45.00%	8.57%	45.00%	8.57%	51.43%	15.00%
Deck B	15.00%	51.43%	15.00%	51.43%	8.57%	45.00%
Trial number	70	80	90	90	80	70
**SEQUENCE #2**						
Deck A	45.00%	8.57%	51.43%	15.00%	51.43%	15.00%
Deck B	15.00%	51.43%	8.57%	45.00%	8.57%	45.00%
Trial number	80	70	90	80	70	90

After completing all trials, each subject was asked two multiple-choice questions concerning his/her choice strategy^5^ and how often the deck reward shifted^6^, and one fill-in question about his/her prediction of the total score.

### Data analysis

Differences across the three groups were analyzed using either ANOVA or a priori *t*-tests (whenever appropriate). A *p*-value of <0.05 was considered statistically significant. Note that while the summary statistics of total scores provide a first glimpse of how group performance might differ, it says very little about the reward sensitivity that is one of the key features testable by the DRT design. In the literature, a so-called “(generalized) matching law” has been used to quantify the sensitivity of performance (of choosing the advantageous option) in reinforcement learning tasks. Accordingly, as a next-step analysis, we performed the matching law analysis to assess the relationship between choice allocation and reward received. Further, to help explain the task performance in the DRT, it is desirable to fit the trial-by-trial choice behavior with a standard reinforcement learning model. We also computed the correlations of the estimated parameter values and the PANSS p1 + p3 subscores using Pearson's correlation coefficient. The impact of the parameters in the model on the overall performance was evaluated by simulation. We now briefly describe the matching law and the reinforcement learning model.

#### Matching law

The *matching law*, first characterized by Herrnstein (1961) and later generalized by Baum (1974), refers to the regularity in data between choice behavior and reward received in reinforcement learning initially observed in animal studies. In some perspective, matching law plays a role similar to Weber's law in psychophysics; both are empirical “laws” that capture certain regularities of data. To examine whether subjects in the three groups distributed their choice frequencies between the two decks (denoted by C_A_ and C_B_, respectively) in agreement with the respective reward frequencies received (R_A_ and R_B_), we applied Equation (1), which is the (generalized) matching law (Baum, 1974), for each block:

(1) $$\log_{2}\left(\frac{{\text{C}}_{\text{A}}}{{\text{C}}_{\text{B}}}\right)=s\log_{2}\left(\frac{{\text{R}}_{\text{A}}}{{\text{R}}_{\text{B}}}\right)+\log_{2}\text{k}.$$

The slope *s* is interpreted as the *sensitivity* of choice allocation in response to reward frequency, and can be used to indicate the overall consistency of choice behavior of choosing the advantageous deck.

#### The reinforcement learning model

Since our main goal is to disentangle the two components (i.e., belief formation and belief perseveration) from the DRT data, we further fitted trial-by-trial choice data using a standard reinforcement learning (RL) model (also called *Q-learning*) under the *temporal difference* learning framework (Watkins and Dayan, 1992; Sutton and Barto, 1998).^7^ This model comprises two parts, the value-updating rule and the choice rule. The value-updating rule specifies how the expectation for one deck is updated on each trial. We use deck A as an example:

(2) $${\text{Q}}_{\text{A}}\left(\text{t}+1\right)={\text{Q}}_{\text{A}}\left(\text{t}\right)+\alpha \left({\text{R}}_{\text{A}}(\text{t})-{\text{Q}}_{\text{A}}(\text{t})\right),$$

where Q_A_ (t) is the expected value and R_A_ (t) is the actual reward on trial *t*. Note that R_A_ (t) − Q_A_ (t) is the RPE, which represents the discrepancy between the expected reward and the reward just received on trial *t*. The key to maximizing the speed of learning from the RPE for this value-updating rule is the parameter α, which represents the *learning rate* that determines how quickly the estimation of an expected value is updated from the trial-by-trial feedback of the prediction error. At the beginning of the task, the expected values of decks A and B were set to zero.

Reinforcement learning also requires a balance between *exploration* (here, “inquiring” into the seemingly disadvantageous option) and *exploitation* (here, “clinging” to the seemingly advantageous option) (Daw et al., 2006). For the choice rule of the model, it is common to assume that the probability of choosing each deck is determined by the so-called “softmax” rule, a formulation consistent with the ratio-scale representation derived from Luce's choice axiom (Luce, 1959) in the mathematical psychology literature. This formulation takes a logistic form. Taking deck A as an example, we have:

(3) $${\text{P}}_{\text{A}}\left(\text{t}+1\right)=\frac{{\text{e}}^{\beta {\text{Q}}_{\text{A}}(\text{t})}}{{\text{e}}^{\beta {\text{Q}}_{\text{A}}(\text{t})}+{\text{e}}^{\beta {\text{Q}}_{\text{B}}(\text{t})}},$$

where the parameter β represents the *choice perseveration*, a term referring to the tendency to take actions based on the expected reward values. For our exemplary formulation in Equation (3), a large value of β means that participants have a higher degree of exploitation of the expected reward value of Deck A, and a zero value of β indicates that participants choose the two decks at random.

The parameters α and β in the RL model were estimated using a hierarchical Bayesian estimation method, which was recently advocated by some researchers (e.g., Lee, 2011) and has been used for fitting of similar RL models (Wetzels et al., 2010). The hierarchical layout of estimation followed closely the graphical Bayesian modeling approach described in Lee and Wagenmakers (2013). We used WinBUGS (Lunn et al., 2000) to approximate the posterior distributions of parameters using the Markov Chain Monte Carlo technique. Three chains were used, and each chain contained 28,000 iterations. The first 8000 samples were deleted, and we took samples at an interval of 5. Thus, a total of 12,000 samples were used for the estimate of each posterior parameter distribution.

Parameters between any two groups were compared by computing the difference between the values of the two posterior distributions in each run obtained from the hierarchical Bayesian estimation. By checking whether the probability of the posterior distribution of differences is greater (or less) than zero, one can evaluate the strength of evidence for differences in group-mean parameters. Alternatively, one can use the Bayes factor (BF), an odd ratio of marginal likelihood of the two models (or hypotheses) of interest, to index the evidence strength of the alternative hypothesis against the null hypothesis (Kass and Raftery, 1995). A large BF value (>3) would (at least) “positively” favor the alternative hypothesis and a BF value between 1 and 3 would “weakly” favor the alternative hypothesis. To evaluate the differences of group-mean parameters, in this study we also used a method based on the Savage-Dickey density ratio (see Wagenmakers et al., 2010, for an introduction) to compute the BF values.

## Results

### Behavioral data

**Figure 2:**
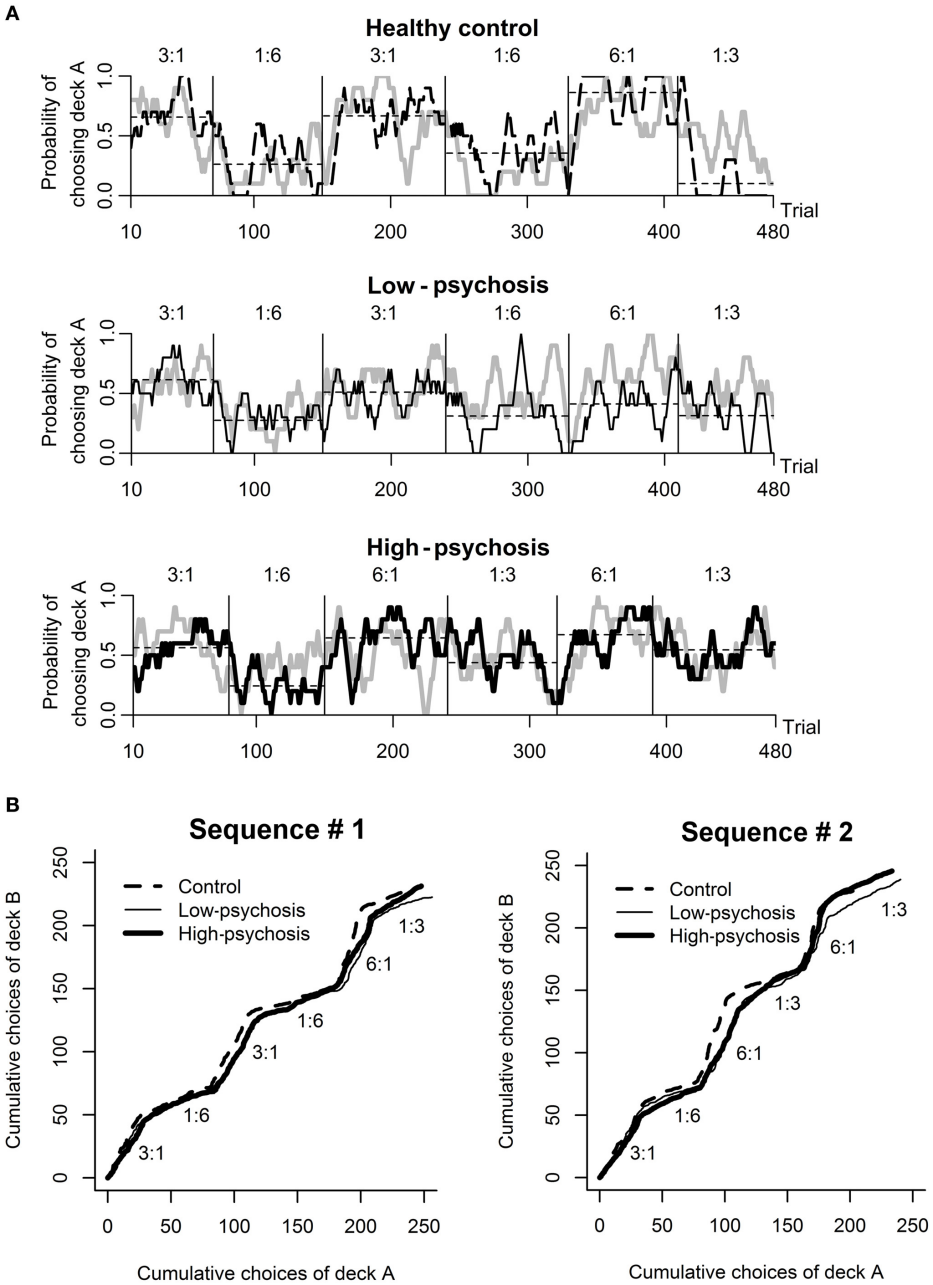
**Behavioral performance in the dynamic reward task**. **(A)** An illustration of the time course of the observed (black) and predicted (gray; drawn based on the best-fitting RL model) choice behavior for one subject from each of the three groups (from top to bottom panels: healthy control, low-psychosis, and high-psychosis groups) in the testing session. Each of the curves was smoothed with a 10-trial moving average. The horizontal thin dashed line shows the average choice within each block for that subject. **(B)** The time course of the average choice pattern for each of the three groups in each of the two sequences (#1 and #2) of probability assignment used in the testing session. The numbers above each block indicate the ratio of assigned reward probability.

Analysis of the behavioral data from the DRT revealed that most subjects in each of the three groups chose the higher reward probability deck more than 50% of the time in the training session (high-psychosis: 26 of 26; low-psychosis: 18 of 19; control: 22 of 24), and all subjects correctly identified the advantageous deck. For the testing session, we observed that SZ patients in the high- and low-psychosis groups generally showed more variation in choice behavior across trials than the control group. To illustrate, we depicted (in black) in Figure 2A the time courses of observed choice behaviors for one subject from each of the three groups. For each exemplary subject, we also display the predicted curve (gray) computed from the best-fitting RL model for comparison. Visual inspection suggests that the patterns of the observed and predicted curves were rather consistent in each case.

The time course of the group-level choice behavior of each of the three groups under each of the two probability-assignment sequences is shown in Figure 2B, from which it is evident that subjects' average choice behavior in each block was largely consistent with the scheduled probability assignment of reward to that block. The average total scores among the three groups were not significantly different [*F*_(2, 66)_ = 0.97, *p* = 0.38; high-psychosis: *M* = 168.2, *SD* = 16.5; low-psychosis: *M* = 171.7, *SD* = 14.9; control: *M* = 174.5, *SD* = 16.1].

Regarding the questionnaires requested for all subjects after the testing session, we found that the answers from the three groups were not different for the first two questions (namely, the choice strategy and how often the deck reward shifted). For the third question (namely, the prediction of the total score), however, the average predicted scores among the three groups were significantly different [*F*_(2, 66)_ = 4.34, *p* = 0.02; high-psychosis: *M* = 127.4, *SD* = 78.9; low-psychosis: *M* = 155.1, *SD* = 77.1; control: *M* = 91.7, *SD* = 55.0]. Comparing the average predicted and actual scores for each subject, we found that there was a trend of underestimation of performance in all three groups. Especially, for the controls and the high-psychosis group the differences were statistically significant [*t*_(23)_ = 8.7, *p* < 0.001 and *t*_(25)_ = 2.63, *p* = 0.01, respectively], indicating that subjects underestimated the potential reward points they would obtain. It remains an open question whether this pattern is typical to this kind of task and/or reflects certain characteristic of the groups.

### Matching law analysis

As mentioned previously, the matching law is more appropriate for uncovering the possible difference of group performance in terms of reward sensitivity. Using least-squares regression, we fit Equation (1) to data from the *steady states* of the DRT, defined as Trials 21–70 in each block. The blocks in which subjects gained no reward for either of the two decks (i.e., R_A_ or R_B_ = 0) were excluded from analysis.

**Figure 3:**
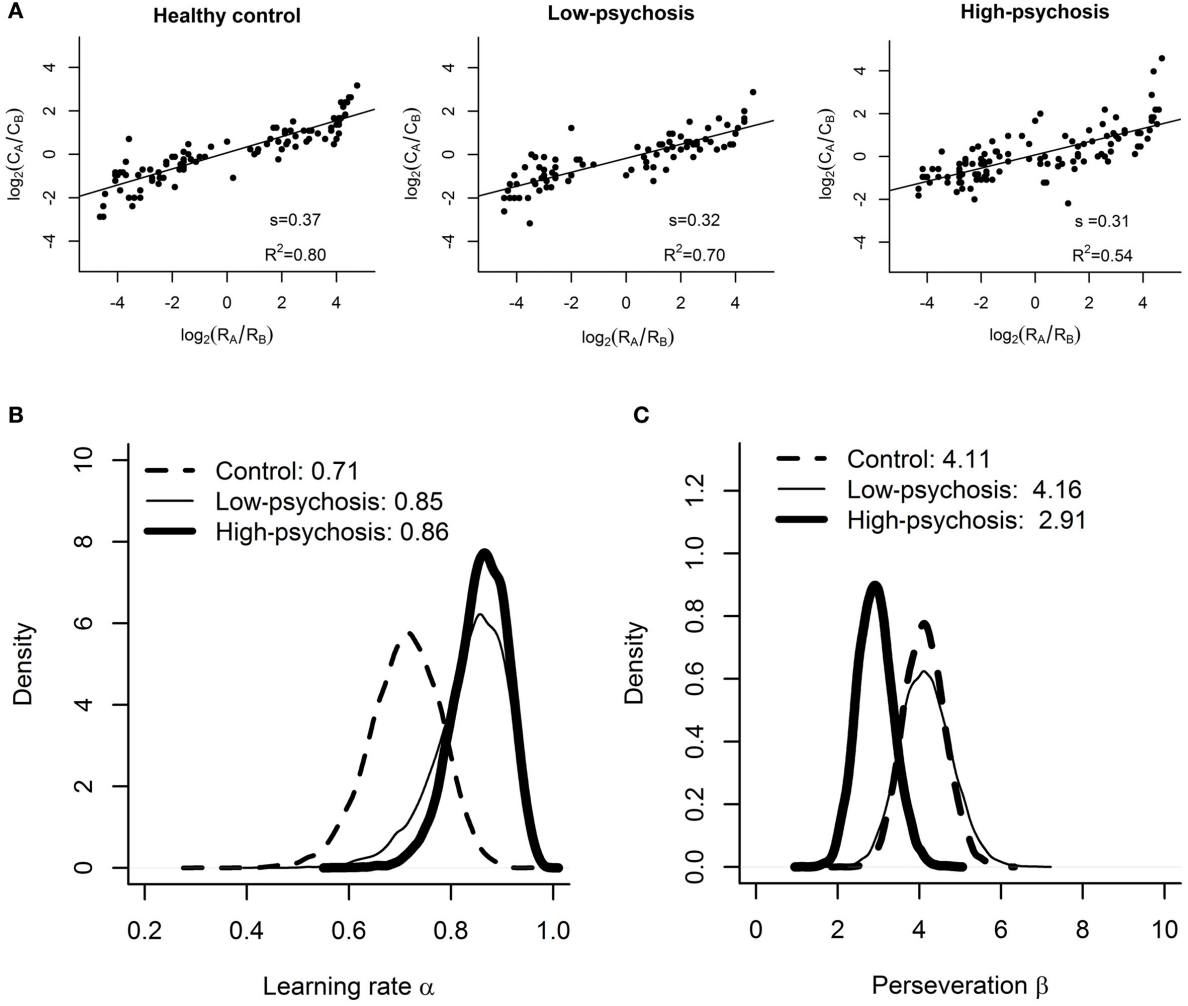
**Results of the matching law analysis and RL model fitting**. **(A)** Estimated values of reward sensitivity *s* in the matching law for the control, low-psychosis, and high-psychosis groups (from left to right panels). Group-level posterior distributions of the **(B)** learning rate α and **(C)** choice perseveration β for the control (dashed curve), low-psychosis (solid, thin curve), and high-psychosis (solid, thick curve) groups.

As depicted in Figure 3A, the matching law analysis showed that the estimated values (± standard errors) of reward sensitivity *s* for the control, low-psychosis, and high-psychosis groups were 0.37 (± 0.02), 0.32 (± 0.02), and 0.31 (± 0.03), respectively, indicating an “undermatching” pattern in all three groups. One-tailed a priori *t*-tests revealed that the values of reward sensitivity for the low- and high-psychosis groups were both significantly lower than that for the control group [*t*_(188)_ = 1.7, *p* = 0.05, and *t*_(219)_ = 1.9, *p* = 0.03, respectively], indicating that SZ patients were less adept at allocating their choice behavior in accord with the reward frequencies that they had experienced. Furthermore, the *R*^2^ values for the control, low-psychosis, and high-psychosis groups were 0.80, 0.70, and 0.54, respectively, indicating a gradual decline in the correlation of choice behavior with reward frequency that was dependent on the severity of positive psychotic symptoms.

### Fitting of the reinforcement learning model

For the learning rate α, the posterior sample means and their 95% credible intervals (*CI*) for the control, low-psychosis, and high-psychosis groups were 0.71 (*CI* = (0.56, 0.84)), 0.85 (*CI* = (0.68, 0.94)), and 0.86 (*CI* = (0.74, 0.94)), respectively (see Figure 3B). The posterior distribution of group mean differences of the parameter α between the control group and the high-psychosis (low-psychosis, respectively) group showed a 0.039 (0.093, respectively) probability of being greater than zero, providing marginal to moderate evidence favoring the claim that the learning rate of the control group was lower than those of both SZ groups. This conclusion is also supported by the Bayesian hypothesis test; we obtained *BF* = 2.95 (*BF* = 1.66, respectively), slightly in favor of the evidence that the learning rate in the high-psychosis (low-psychosis, respectively) group is larger than that in the control group.

For the choice perseveration β, the posterior sample mean for the control group was 4.11 (*CI* = (3.15, 5.17)), which was similar to that for the low-psychosis group 4.16 (*CI* = (2.98, 5.47)). The two estimated values, however, were much larger than the estimate of 2.91 for the high-psychosis group (*CI* = (2.09, 3.83)) (see Figure 3C). The posterior distribution of group mean differences of the parameter β between the high-psychosis group and the control (low-psychosis, respectively) group indicated a 0.034 (0.055, respectively) probability of being greater than zero, providing moderate evidence favoring the claim that the high-psychosis group exhibited a lower degree of choice perseveration (or exploitation) than the control and low-psychosis groups. The Bayes factor *BF* = 2.11 (*BF* = 2.22, respectively) for testing the hypothesis that choice perseveration is higher in the control (low-psychosis, respectively) group than in the high-psychosis group also supported the claim.

The distinction of estimates of choice perseveration between the two SZ groups was further evaluated by correlating all patients' estimated parameter values of perseveration with their PANSS p1 + p3 scores, with the units of the different medication dosages normalized in analysis. We found a (partial) correlation of −0.26, which was marginally significant (*p* = 0.09), indicating that our assessment of the SZ patients' degree of exploitation may, to some extent, reflect the severity of their positive psychotic symptoms. On the other hand, no significant correlation (*r* = −0.04, *p* = 0.79) was found between the estimated values of the learning rate and the PANSS p1 + p3 scores, indicating that our hypothesis about the association between the learning rate and the severity of the positive psychotic symptoms of the SZ patients is not supported.

### Simulation: the impact of the parameters in the RL model on the performance

**Figure 4:**
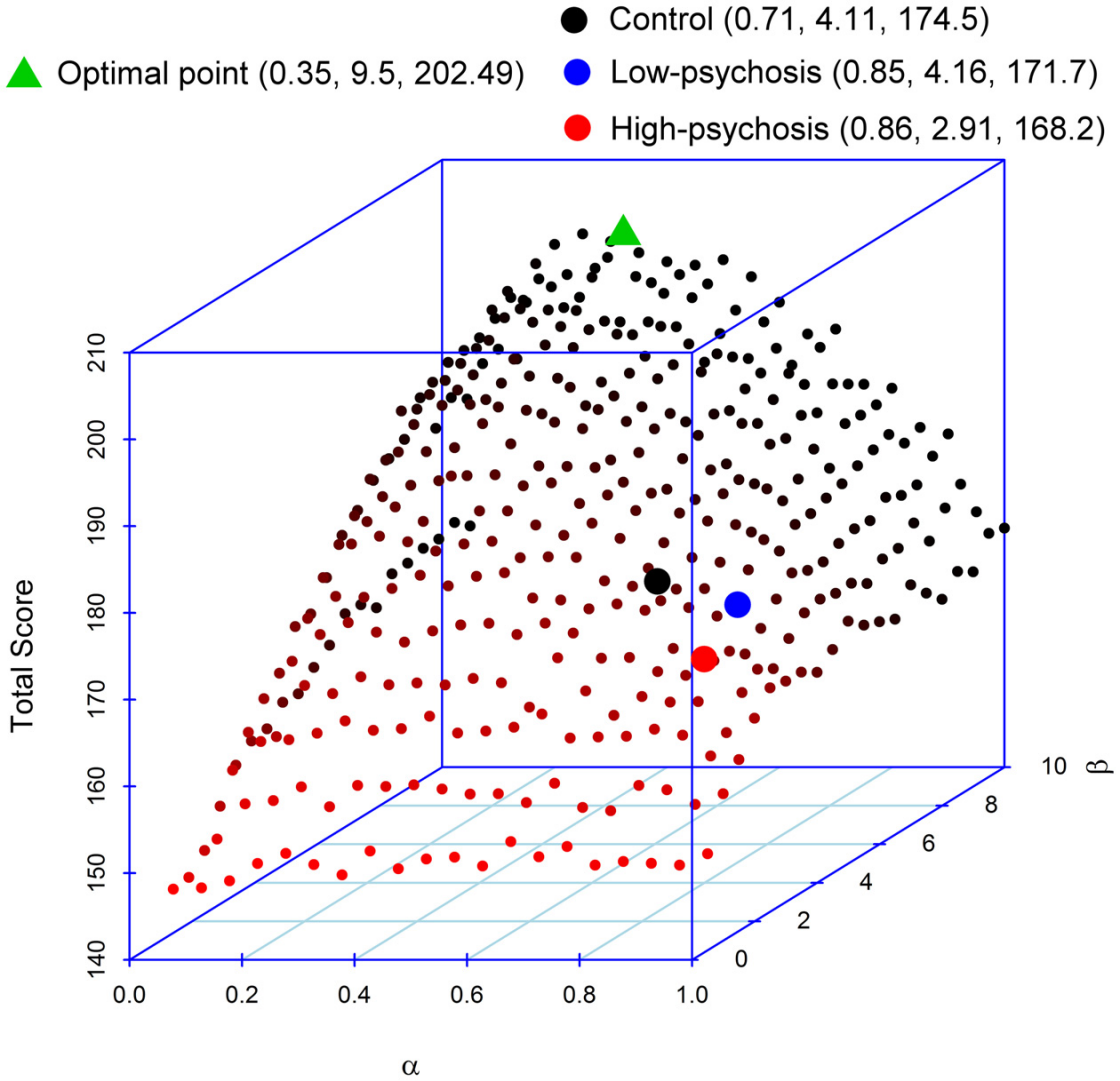
**Impacts of parameters α and β on the total score of the dynamic reward task**. The averaged estimated parameters and the total score of controls and both SZ groups are marked by the three filled circles. We also mark the position of the maximal point obtained from the simulation. The respective coordinate values of α, β, and total score are indicated in parentheses in the figure legend.

As mentioned earlier, reinforcement learning requires a balance between updating (for belief formation) and exploitation (for belief perseveration). Indeed, high learning rates do not imply optimal task performances. To illustrate this point, we performed a simulation to evaluate how the two parameters α and β in the RL model affect performance in the DRT. In the simulation, we paired the α-values (from 0.05 to 1, in an increment of 0.05) with the β-values (from 0.5 to 10, in an increment of 0.5) such that there were a total of 400 pairs in the setting. We then inserted each pair of parameter values into the model to simulate the data. We repeated the procedure 100 times. Figure 4 displays the simulated average total scores, with the standard deviations ranging from 9.59 to 15.63, obtained from each of the 400 pairs of parameters. The result indicates that optimal performance (in terms of maximizing the total point) occurs when the α-value is about 0.35. Performance decreases as the α-value moves away from 0.35. Thus, changing beliefs too fast (after experiencing a limited number of trials) might not be a good strategy for reinforcement learning. Further, Figure 4 shows that the optimality of performance is modulated by the β-value that more perseveration results in better performance. We found that when the α-value is within the range of 0.2–0.45, most of the high scores occur when the β-value is above 7. In our experiment, the averaged estimated values of α (and β, respectively) for both SZ patients and controls were all larger than 0.35 (and smaller than 7, respectively) (see the three filled circles in the figure), indicating deviations from optimal performance. In particular, compared with the control group, the α-values for both high- and low-psychosis SZ patients were less optimal, and the β-value for the high-psychosis group was relatively far away from the optimal value.

## Discussion

In this study, we developed a computerized version of the DRT and accompanied it with a standard RL model to examine the relationship between the RPE process and the psychotic symptoms (as revealed by the scores of the p1 “delusion” and p3 “hallucinatory behavior” subscales in the PANSS) of SZ patients. In particular, the implicit switching of the reward probabilities associated with each of the decks in the experimental sequence allows one to test whether and how efficiently the subjects learn to adjust their decisions based on feedback. Matching law analysis revealed that both psychosis groups exhibited reduced reward sensitivity than healthy controls. We further fit the DRT data with a standard RL model and found that, on average, SZ patients had higher learning rates than healthy controls and that the degree of perseveration in choice appeared to be negatively correlated (*p* = 0.09, trending toward significance) with the severity of positive psychotic symptoms.

Whether positive or negative symptoms of schizophrenia are more related to the dysfunction of RPE signaling is still under debate in the literature (Corlett et al., 2007; Murray et al., 2008; Kasanova et al., 2011; Strauss et al., 2011; Deserno et al., 2013). To take a glimpse of this issue, we also correlated SZ patients' scores on the negative-symptom subscales of the PANSS with their estimated parameter values in the RL model. We found no significant results for any of the parameters (the correlation was 0.02 (*p* = 0.92) for the learning rate parameter and was −0.13 (*p* = 0.40) for the choice perseveration parameter), suggesting that for the DRT in which the decision-making process involves unitary reward but not punishment, dysfunction of RPE signaling is more associated with the positive symptoms of psychosis.

The use of the DRT provides several advantages. Especially, the task is simple and can be completed within 20 min, and thus has the potential to be conducted in clinical groups. Further, the task can be easily adapted for combination with a variety of cognitive and imaging technologies, such as fMRI, PET, ERP, and MEG. We also have shown that through matching law analysis as well as fitting to trial-by-trial DRT data with a standard RL model, sensitivity and reward learning can be estimated. Importantly, both learning rate and choice perseveration, which usually cannot be inferred from conventional analyses of behavioral data, can be extracted (here, using the Bayesian estimation approach). These new measures might be a starting point for future studies aiming to develop sensitive markers that predict early on the progression of the disease and the response to treatment. Thus, accompanied by computational analyses, the DRT provides an alternative for studying reward-related learning and decision making in basic and clinical sciences.

RL models have been increasingly applied to study reward-based learning in humans, non-human primates, and mice (Juckel et al., 2006; Rutledge et al., 2009; Chen et al., 2012). During reinforcement learning, the firing of dopaminergic neurons has been found to correlate with the characteristics of prediction errors postulated in the RL models (Schultz et al., 1997; Montague et al., 2004; Glimcher, 2011), supporting the dopamine reward prediction error hypothesis (Glimcher, 2011). In the present study, we recapitulated the dynamics of RPE from the DRT data of SZ patients through fitting of a standard RL model, and our findings suggest (though indirectly) that abnormal RPE processes tend to correlate with sub-optimal performances in reinforcement learning that might be related to psychotic experiences and aberrant dopamine activities.

Finally, since all SZ patients in our study were on antipsychotic medication, some of our findings should be interpreted with caution. Our experimental design only ruled out the dosage difference of antipsychotic medication between the high- and low-psychosis groups (see the last paragraph in section Participants). For those SZ patients we also found no association between the adjusted drug dose and any of the two parameters in the RL model (the correlation was 0.12 (*p* = 0.42) for the learning rate parameter and was -0.1 (*p* = 0.53) for the choice perseveration parameter). Still, it is plausible that medication is a confounding factor that could also explain the performance difference between the SZ patients and controls. Thus, it will be highly interesting to recruit SZ patients who have not started antipsychotic treatment to perform the DRT and compare the results with their performance after beginning medication. Future research along this line would be timely and worthwhile.

### Conflict of interest statement

The authors declare that the research was conducted in the absence of any commercial or financial relationships that could be construed as a potential conflict of interest.

## Footnotes

^1^But see a series of study by Berridge (2007, 2012) about the distinct roles of dopamine on salience and on prediction error signals.

^2^See, however, evidence from other studies (e.g., Kasanova et al., 2011; Strauss et al., 2011) showing that dysfunction of RPE signaling is more associated with the negative symptoms of schizophrenia.

^3^Whether the positive- or negative-symptom subscales of the PANSS correlate more with the DRT-elicited RPE signaling will be briefly discussed in the Discussion section.

^4^There were a total of four sequences if we consider balancing the A, B decks regarding the gain ratio assignment.

^5^The choice options are (1) Fixed on a deck, (2) Fixed on a deck and changed to another deck sometimes, (3) Chose the two decks alternatively, (4) Chose randomly, (5) None of the above.

^6^The choice options are (1) 0 times, (2) 1–3 times, (3) 4–10 times, (4) 11–20 times, (5) 21–30 times.

^7^It is granted that other computational models with more sophisticated mechanisms (see Niv, 2009, for an introduction) may provide more detailed explanations of probability-learning data. Given the simplicity of the DRT design (on unitary reward, with switches of reward probabilities of the two decks across blocks), the standard RL model seemed adequate (as an approximation) to address the issue raised in this study. Thus we did not pursue other models.
